# METTL3 regulates TFRC ubiquitination and ferroptosis through stabilizing NEDD4L mRNA to impact stroke

**DOI:** 10.1007/s10565-024-09844-x

**Published:** 2024-02-02

**Authors:** Wenjie Su, Xiang Yu, Shan Wang, Xu Wang, Zheng Dai, Yi Li

**Affiliations:** 1 Department of AnesthesiologySichuan Provincial People’s Hospital, University of Electronic Science and Technology of China, Chengdu, 610072 Sichuan China; 2 Department of RadiologySichuan Provincial People’s Hospital, University of Electronic Science and Technology of China, Chengdu, 610072 Sichuan China; 3 Department of Echocardiography & Noninvasive Cardiology Laboratory, Sichuan Provincial People’s Hospital, University of Electronic Science and Technology of China, Chengdu, 610072 Sichuan China; 4 No. 2 Ward of Hepatobiliary Surgery, Sichuan Provincial People’s Hospital, University of Electronic Science and Technology of China, Chengdu, 610072 Sichuan China; 5 Emergency Department, Sichuan Provincial People’s Hospital, University of Electronic Science and Technology of China, No. 32 West Second Section, First Ring Road, Chengdu, 610072 Sichuan China

**Keywords:** Stroke, Ubiquitination, Ferroptosis, METTL3, NEDD4L

## Abstract

**Background:**

Stroke is a major medical problem, and novel therapeutic targets are urgently needed. This study investigates the protective role and potential mechanisms of the N6-methyladenosine (m6A) RNA methyltransferase METTL3 against cerebral injury resulting from insufficient cerebral blood flow.

**Methods:**

In this study, we constructed mouse MCAO models and HT-22 cell OGD/R models to mimic ischemic stroke-induced brain injury and neuronal damage. We generated NEDD4L knockout and METTL3 overexpression models and validated therapeutic effects using infarct volume, brain edema, and neurologic scoring. We performed qRT-PCR, western blotting, and co-immunoprecipitation to assess the influence of NEDD4L on ferroptosis markers and TFRC expression. We verified the effect of NEDD4L on TFRC ubiquitination by detecting half-life and ubiquitination. Finally, we validated the impact of METTL3 on NEDD4L mRNA stability and MCAO outcomes in both in vitro and in vivo experimental models.

**Result:**

We find NEDD4L expression is downregulated in MCAO models. Overexpressing METTL3 inhibits the iron carrier protein TFRC by upregulating the E3 ubiquitin ligase NEDD4L, thereby alleviating oxidative damage and ferroptosis to protect the brain from ischemic injury. Mechanistic studies show METTL3 can methylate and stabilize NEDD4L mRNA, enhancing NEDD4L expression. As a downstream effector, NEDD4L ubiquitinates and degrades TFRC, reducing iron accumulation and neuronal ferroptosis.

**Conclusion:**

In summary, we uncover the METTL3-NEDD4L-TFRC axis is critical for inhibiting post-ischemic brain injury. Enhancing this pathway may serve as an effective strategy for stroke therapy. This study lays the theoretical foundation for developing m6A-related therapies against ischemic brain damage.

**Supplementary Information:**

The online version contains supplementary material available at 10.1007/s10565-024-09844-x.

## Introduction

Ischemic stroke is a prevalent neurological condition resulting from the constriction or blockage of cerebral blood flow, leading to extensive damage of brain tissue and significant neurological impairments (Zhang et al. 2021). The condition is characterized by a debilitating nature, accompanied by a high incidence of disability, recurrence, and mortality, thereby posing a significant threat to human health. Despite notable advancements in the identification and management of ischemic stroke, the outcomes continue to be unsatisfactory (Tuo et al. 2022). Currently, thrombolysis remains the sole approved intervention for acute ischemic stroke; however, the efficacy of this treatment is limited due to its narrow focus and the potential risks associated with hemorrhagic transformation. (Liu et al. 2020; Stockwell et al. 2017). Therefore, conducting comprehensive research on the pathological mechanisms and treatment strategies of ischemic stroke is imperative in order to identify novel neuroprotective agents.

Recent research has unveiled the pivotal involvement of ferroptosis, a form of programmed cell death, in the progression and advancement of ischemic stroke. Consequently, targeting ferroptosis has emerged as a promising therapeutic strategy for this specific condition (Cui et al. 2021; Liu et al. 2021). This strategy involves the use of agents that inhibit or promote ferroptosis pathways to prevent or induce neuronal death, respectively (Chen et al. 2021a). Further investigation is imperative to elucidate the specific molecular pathways implicated in ferroptosis and to devise efficacious neuroprotective agents. The role of iron overload as a significant contributor to ferroptosis following ischemic stroke has been identified (Fu et al. 2022). Elderly stroke patients tend to have a poor prognosis, and research findings have indicated that excessive iron deposition in the brain may exacerbate ferroptosis in neurons after post-ischemic brain injury (Guo et al. 2023). The presence of elevated ferritin levels in the bloodstream has been associated with an unfavorable prognosis among individuals suffering from acute cerebral infarction.. In animal models replicating ischemic stroke through middle cerebral artery occlusion (MCAO), deposition of iron has been observed within the ischemic region of brain tissue (Mao et al. 2022). The ischemic brain tissue demonstrates a significant increase in iron deposition due to disruption of the blood-brain barrier (BBB) and subsequent heightened permeability. (Weiland et al. 2019). Consequently, a substantial quantity of ironates the highly permeable blood–brain barrier (BBB) and infiltrates the brain parenchyma, thereby facilitating the generation. Ultimately, this process leads to damage in nucleic acids, proteomes, and membranes while triggering cell death associated with ferroptosis (Ma et al. 2022). Studies have found that the iron chelator, deferoxamine (DFO), is safe and well-tolerated in the treatment among patients afflicted with ischemic stroke, which possesses the potential to reduce iron saturation of blood transferrin, decrease peroxide levels in serum, and augment the capacity for trapping free radicals as potent antioxidants (TRAP) (Yu et al. 2021). DFO may have anti-oxidative stress effects and play a neuroprotective role in stroke patients, thereby improving their prognosis (Yuan et al. 2021).

Ubiquitination is a common epigenetic modification that holds a pivotal role in the degradation of target proteins through the proteasome pathway, regulation of protein transport, regulation of enzyme activity, and assembly of protein signaling complexes (Nakka and Mohammed 2020). Ubiquitination is in turn catalyzed by three key enzymes: ubiquitin activating enzyme (E1), ubiquitin conjugating enzyme (E2), and ubiquitin ligase (E3). Among them, E3 ubiquitin ligases can directly bind to target proteins and have substrate specificity, which is considered to be the most critical component of the ubiquitinated protein modification mechanism (Wang et al. 2021). E3 ubiquitin ligases have been implicated in the direct modulation of cancer initiation and progression (Xu et al. 2022). NEDD4L, also known as RSP5, PVNH7, NEDD4-2, NEDD4.2, and hNEDD4-2, is situated at 18q21.31 and is a constituent element of the HECT E3 ligase family. Studies have demonstrated that NEDD4L functions as an E3 ubiquitin ligase in various cancers by promoting the ubiquitination of target proteins cancer cell proliferation, apoptosis, epithelial-mesenchymal), cancer stem cells, and chemotherapy resistance (Donovan and Poronnik 2013). The co-expression of NEDD4 has been demonstrated to elicit an upregulation in the expression of Ndfip1, which has been identified as conferring neuroprotective effects in instances of traumatic brain injury. (Oliver et al. 2006). Furthermore, increased expression of it exhibits a strong correlation with neuronal survival following ischemic stroke. These findings suggest that the regulation of neuronal injury after stroke may involve the expression of NEDD4L, although the specific pathway and underlying molecular mechanism remain elusive (Lackovic et al. 2012). Therefore, the primary aim of this research is to investigate the regulatory mechanism underlying NEDD4L in stroke-induced ferroptosis. This investigation holds great potential for identifying novel therapeutic targets with implications for stroke treatment.

## Materials and methods

### Bioinformatics analysis

In this study, the BIOGRID database (https://thebiogrid.org/) was used to screen the proteins that interacted with NEDD4L (Oughtred et al. 2021). The Ubibrower database (http://ubibrowser.bio-it.cn/ubibrowser/) was used to screen potential ubiquitinated E3 ligases of TFRC (Li et al. 2017). In the m6A analysis, RM2Target database (http://rm2target.canceromics.org/#/citation) had been employed for analyze the RNA methylation correlation analysis of potential binding of NEDD4L (Bao et al. 2023), and the SRAMP database (http://www.cuilab.cn/sramp) had been employed to predict the RNA binding targets of NEDD4L and draw the secondary structure (Zhou et al. 2016).

### Animals and middle cerebral artery occlusion (MCAO) model

All C57BL/6 mice, aged 8–10 weeks, were purchased from Beijing Charles River Laboratory Animal Company and housed in a pathogen-free facility under a 12-h light/dark cycle with ad libitum access to food and water. All animal studies were performed in accordance with the Ethical Guidelines for the Use and Care of Laboratory Animals and were approved by the Animal Ethics Committee of Sichuan Provincial People’s Hospital.

All mice were fasted and water deprived for 12 h. MCAO model was established by internal carotid artery occlusion method. The mice were intraperitoneally injected with pentobarbital at a dosage of 60 mg/kg to induce anesthesia and subsequently positioned in the prone stance for stabilization. The neck was disinfected, and a 1.5-cm incision was cut located at the midpoint of the cervical region. The subcutaneous tissue, glands, and muscles were separated, and the right common carotid artery and internal carotid artery were exposed.

A knot was tied at both ends of the external carotid artery, a small incision was cut between the two knots, a thread plug was inserted, and the external carotid artery was pulled, so that the thread plug slowly entered the internal carotid artery about 10 mm or so if slight resistance was encountered, the entry was stopped. The thread plug was securely fastened, followed by the suturing of the incision in a layered manner. Furthermore, appropriate disinfection measures were implemented on the thread plug to effectively mitigate any potential risk of infection. Mice underwent reperfusion after 90 min of occlusion. In the sham operation group, the operation was the same as tMCAO, but the suture plug only penetrated about 5 mm, without causing cerebral ischemia.

### Construction of NEDD4L knockout mice

NEDD4L conditional knockout mice were purchased from Guangzhou Cyagen Biosciences. To generate NEDD4L knockout mice, we designed a guide RNA targeting exon 7 of NEDD4L and cloned it into the PX459 vector. Mouse embryonic stem cells were transfected with this construct, and single cell clones were isolated. These clones were screened by PCR amplification and sequencing of the target region to identify mutant alleles. Southern blot analysis further validated NEDD4L disruption in positive clones. The appropriately directed embryonic stem cells were subsequently introduced into mouse blastocysts and implanted into surrogate mothers to generate chimeric progeny. Chimeric mice were bred with wildtype mice to achieve germline transmission of the NEDD4L mutation. Intercrosses between NEDD4L heterozygotes resulted in homozygous knockout mice. Genotyping confirmed the absence of wildtype NEDD4L alleles, and western blotting verified deletion of NEDD4L protein in the homozygous mice (Gao et al. 2021).

### *In vitro* cell culture, oxygen–glucose deprivation/reperfusion (OGD/R)

The OGD/R assay was utilized for the in vitro experiments. In brief, mouse hippocampal (HT-22) cells (obtained from Procell Co, LTD) was exposed to 1% oxygen in glucose-free DMEM at 37 ℃ for 4 h for OGD. The O_2_ level in the medium was guaranteed to equilibrate to 1% hypoxia within 2 h under hypoxic conditions. To ensure the effect of hypoxia, an adequate amount of sugar-free DMEM was added to the dishes of the hypoxia workstation for 4 h before OGD. In addition, oxygen–glucose deprivation manipulations were performed in a hypoxic workstation in this study to eliminate oxygen exposure. Throughout this time period, HT-22 cells were cultured in a complete medium and exposed to a humid CO_2_ and 95% air for 8 h prior to collection same washing and medium modifications but were consistently maintained at a temperature of 37 ℃ in a complete medium with 5% CO_2_ and 95% air. The melatonin was initially dissolved in pure ethanol and subsequently diluted to achieve the desired concentrations in the basal medium. In this study, 20 μM and 40 μM of Mel were added 30 min before OGD/R treatment and then treated.

### 2,3,5-triphenyl tetrazolium chloride (TTC) assay

After MCAO operation for 48 h, slowly separate the skull base tissue to obtain complete brain tissue, put it on the mold, and cut it into four coronal slices, each about 2 mm thick. Immerse the brain slices in TTC solution, incubate them at 37 ℃ for 30 min, take out the brain slices, and fix them with paraformaldehyde for 24 h. Scan and image them with a scanner. Normal brain tissues are red areas, ischemic brain tissues are white areas, and the volume of cerebral infarction is measured with Image J software.

### Assessment of stroke injury

The evaluation of each mouse was conducted by three examiners who were blinded to the treatment regimen. Scores range from 0 (no dyskinesia) to 4 (severe). After evaluating neurological deficits, the infarct area was photographed and the wet weight of the brain slices was quantified. The slices were then dried at 105 ℃ for 48 hours to determine their water content. Brain water content was calculated using the formula (wet weight - dry weight) / wet weight × 100%. 

### Cell viability assay

The viability of cells in each treatment group was evaluated using the Cell Counting Kit-8 (CCK-8) assay in this study The supplier’s instructions were meticulously adhered to in order to precisely execute the designated procedures, and subsequent measurement of absorbance at 450 nm was performed for each treatment group.

The proliferation of HT-22 cells was assessed using 5-ethynyl-2′-deoxyuridine (EdU) staining. Various treatments were administered to HT-22 cells, followed by a 2-h incubation with EdU (20 mmol). Subsequently, the cells were treated with 4% paraformaldehyde and left to fix at ambient temperature for a duration of 20 min. The presence of EDU-positive cells in groups was analyzed utilizing Image J.

### Cell transfection

SiRNA (si-NEDD4L, si-METTL3) and overexpressed vectors (NEDD4L, TFRC) were synthesized from Genepharma company (Shanghai, China). The HT-22 cells were planed 24 h before transfection, and 20 ~ 100 nmol siRNA was transfected according to cell tolerance and transfection efficiency. The reaction system was 4 mL serum-free medium, cultured for 8 h, then replaced with fresh medium, and continued culture for 48 h.

### Real-time fluorescence quantitative PCR (qRT-PCR)

Total RNA of samples was extracted by TRIzol method, and cDNA reverse transcription was performed according to Transcriptor First Strand cDNA Synthesis Kit. β-Actin was used as internal reference. Fast Start Universal SYBR Green Master (Rox) was used for quantitative PCR amplification. PCR reaction system was 20 μL, and the reaction conditions were as follows: an initial denaturation at 95 ℃ for a duration of 2 min, followed by subsequent denaturation at 95 ℃ for a period of 15 s. The annealing process was conducted at a temperature of 60 ℃ for a duration of 30 s. This cycle was repeated for a total number of 40 times. ABI 7500 SDS V1.4 software was used to analyze the data, and 2^−ΔΔCT^ was used for relative quantification. All primer sequences were shown in Supplementary Table 1.

### Western blotting (WB)

The BCA method was employed to quantify the protein concentration extracted from RIPA lysate. Polyacrylamide gel was used for electrophoresis. After electrophoresis, the membrane was wet transferred to 0.45 μm PVDF membrane, reconstituted with 5% low-fat milk and incubated for 2 h, followed by overnight incubation at a temperature of 4 ℃ in the presence of primary antibody, washed with TBST (3 times, 10 min/time), selected HRP labeled IgG from the same source as the primary antibody (anti-TFRC, TFRC, 1:1000; anti-NEDD4L, NEDD4L, 1:1000; anti-ACSL4, ACSL4, 1:2000; anti-GPX4, GPX4, 1:2000; anti-FSP1, ab197896, 1:1000; anti-α-tubulin, ab7291, 1:8000) and incubated at room temperature for 1.5 h, TBST washed the membrane (3 times, 10 min/ time), film exposure, development, and fixing.

### Hematoxylin and eosin (H&E) and Nissl staining

Brains were fixed overnight in 4% paraformaldehyde, embedded in paraffin, and serially sectioned, deparaffinized, and hydrated. The tissue samples underwent routine histological examination using a light microscope following staining with either H&E or 0.1% cresyl violet.

### RNA degradation assay

The cells were initially placed in 6 wells of cell plates with a density of 4 × 10^5^ cells per well. The si-NC group and si-METTL3 were created using the same transfection methods mentioned earlier. After incubating for 48 h, actinomycin was added to inhibit RNA synthesis, following established procedures. Additionally, TRIzol was used to extract total RNA at three time points: immediately and after 6 or 12-h period. Subsequently, cDNA was synthesized through reverse transcription using cDNA synthesis kit. Finally, qRT-PCR was used to assess the expression of NEDD4L mRNA.

### TFRC ubiquitination assay

To identify whether TFRC is the substrate of NEDD4L in cells, cell lines were transiently transfected with pcDNA-Flag-NEDD4L plasmid and pcDNA-His-Ub plasmid, respectively, and the cells were lysed 48 h after transfection. The cells were pre-treated with 10 μM MG132 for 4 h prior to sample collection; 1 mg of total protein was used for co-immunoprecipitation, Flag-TFRC was pulled down using anti-Flag affinity gel, and TFRC ubiquitination was detected using anti-Ub antibody in western blot.

### Measurement of lipid ROS

The HT-22 cells were cultured in 96-well plates with glass bottoms at a seeding density of 3000 cells per well prior to treatment. Subsequently, the cells underwent treatment and were stained simultaneously using BODIPY-C11 dye and DAPI. Following removal of any excess dye through washing, digital images of the stained cells were captured using an Olympus laser scanning confocal microscope equipped with a 60 × objective lens.

### Glutathione measurements

The HT-22 cells were cultured in 6-well plates at a seeding density of 5 × 10^4^ cells per well. After subjecting the cells to the indicated treatments for 48 hours, total intracellular glutathione (GSH) was collected and processed using an assay kit obtained from Nanjing, China, in accordance with the manufacturer's instructions. 

### Statistical analysis

The data was analyzed using SPSS 20.0 software, and the experimental results were presented as mean ± standard deviation (mean ± SD). The one-way analysis of variance (ANOVA) was employed to compare three or more groups, followed by the least significant difference (LSD) test for subsequent pairwise comparisons. Independent sample *t* test was used for comparing two groups. The observed difference exhibited statistical significance at *P* < 0.05 level.

## Result

### Expression levels of NEDD4L in MCAO and OGD/R models

**Fig. 1:**
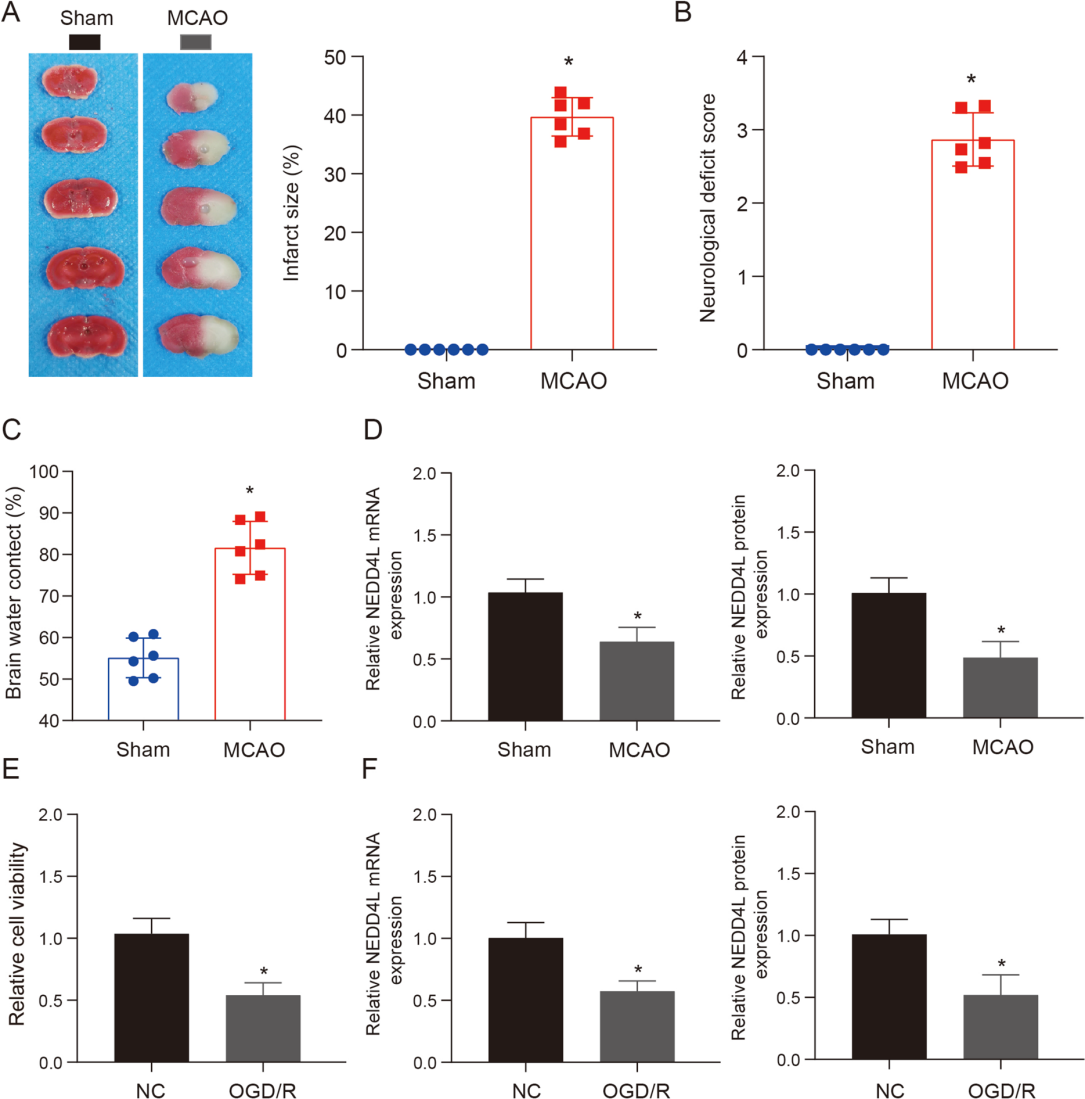
Expression levels of NEDD4L in MCAO and OGD/R models. **A**. The TTC staining technique was employed to identify the ischemic region in mice following MCAO intervention. **B**. Neurological function scores of mice in each treatment group, *n* = 6. **C**. Water content in the brains of mice across different treatment groups, *n* = 6. **D**. QRT-PCR and WB were used to detect the mRNA and protein expression levels of NEDD4L after MCAO treatment, *n* = 6. **E**. CCK-8 was used to detect the cell viability after OGD/R treatment, *n* = 3. **F**. QRT-PCR and WB were used to detect the mRNA and protein expression levels of NEDD4L after OGD/R treatment, *n* = 3. * vs Sham or NC group *P* < 0.05

To confirm the presence of NEDD4L in cases of ischemic stroke, we initially established a mouse model of middle cerebral artery occlusion (MCAO) and subsequently verified the occurrence of ischemic damage (Fig. 1A–C). Further qRT-PCR and WB results showed that NEDD4L mRNA and protein levels were significantly decreased in MCAO group (Fig. 1D). In addition, our results showed that OGD/R treatment could significantly inhibit the cell viability of HT-22 cells (Fig. 1E), and the expression of NEDD4L was found to be significantly decreased in the treatment group (Fig. 1F). These results confirmed our hypothesis and suggested that NEDD4L may contribute significantly to the regulation of stroke.

### Knocking out (KO) the effects of NEDD4L on ischemic stroke injury

**Fig. 2:**
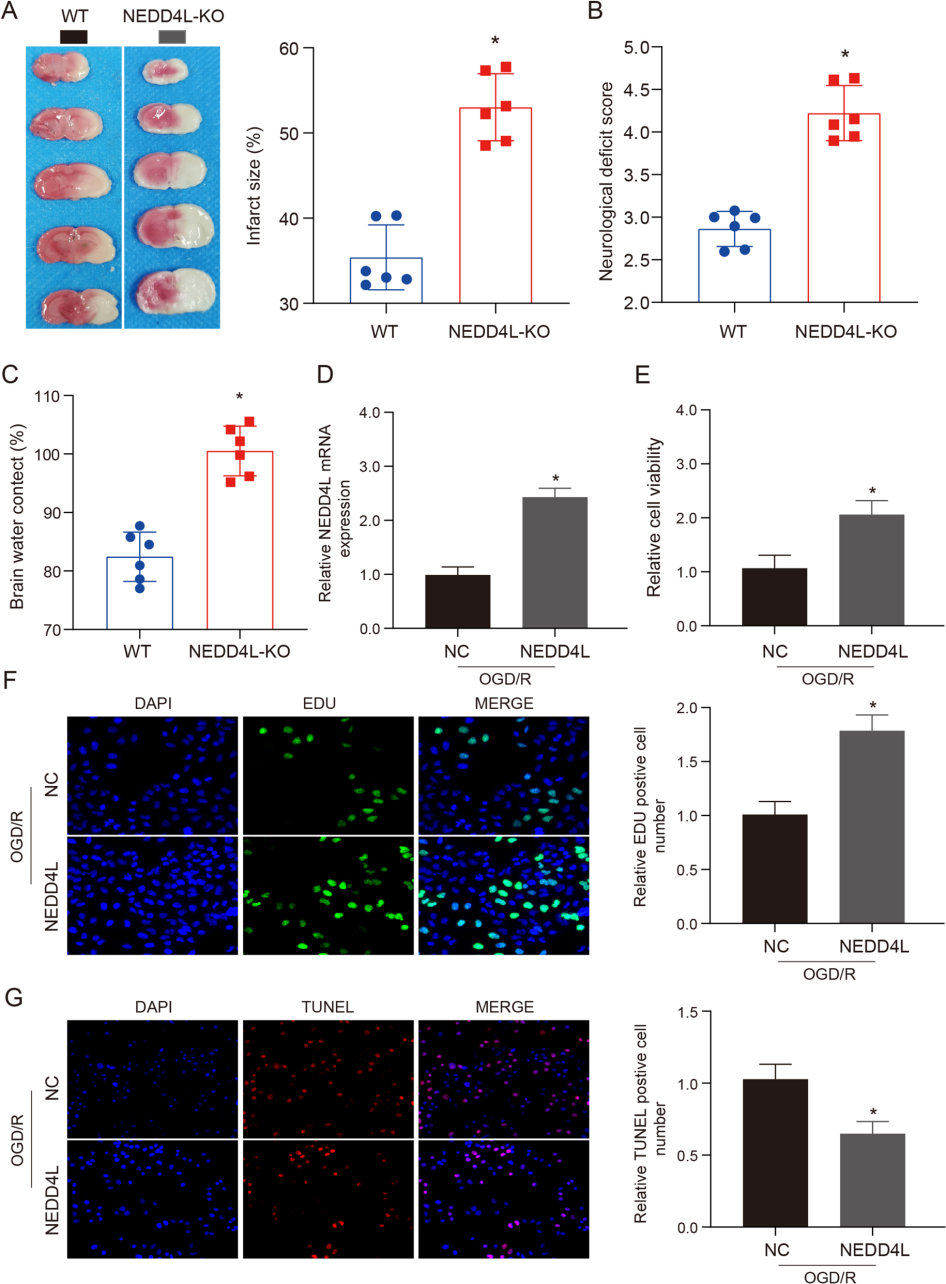
Knocking out (KO) the effects of NEDD4L on ischemic stroke injury. **A**. TTC staining was used to detect the ischemic area of NEDD4L-KO or WT mice after MCAO treatment, *n* = 6. **B**. Neurological function scores of mice in each treatment group, *n* = 6. **C**. Brain water content of mice in each treatment group, *n* = 6. **D**. QRT-PCR was used to detect the mRNA expression levels of NEDD4L after OGD/R or NEDD4L treatment, *n* = 3. **E**. CCK-8 was used to detect the cell viability after OGD/R or NEDD4L treatment, *n* = 3. **F**. The Edu staining was used to detect the cell viability after OGD/R or NEDD4L treatment, *n* = 3. **G**. The TUNEL staining was used to detect the cell apoptosis after OGD/R or NEDD4L treatment, *n* = 3

The E3 ubiquitin ligase NEDD4L has been shown to play a detrimental role in ischemic stroke injury. The aim of this study was to investigate the impact of genetic ablation of NEDD4L on post-ischemic stroke outcomes. We utilized a mouse model with a genetic deletion of NEDD4L and induced focal cerebral ischemia via middle cerebral artery occlusion (Supplementary Fig. 1A). Following stroke, we conducted an assessment of infarct size, neurological deficit, and brain findings which revealed that NEDD4L-KO mice exhibited significantly exacerbated neurological deficit and more pronounced brain edema compared to their wild-type counterparts, indicating a heightened blood-brain barrier impairment (Fig. 2A–C). Furthermore, we constructed a NEDD4L overexpression model in HT-22 cells to examine the effects of NEDD4L overexpression on OGD/R-induced injury (Fig. 2D). We performed EDU staining and CCK-8 assays to assess the proliferative ability of HT-22 cells after OGD/R treatment with or without NEDD4L overexpression (Fig. 2E–F). We found that NEDD4L overexpression significantly improved the cytotoxicity induced by OGD/R. TUNEL staining further revealed that the number of apoptotic cells was markedly decreased in the NEDD4L overexpression group (Fig. 2G). In summary, by generating an NEDD4L-overexpressing HT-22 cell line, we demonstrated that NEDD4L overexpression ameliorated OGD/R-induced cytotoxicity and apoptosis in these cells. Our findings indicate a protective role for NEDD4L against ischemic injury in vitro.

### The regulatory role of NEDD4L in ferroptosis during MCAO and OGD/R models

**Fig. 3:**
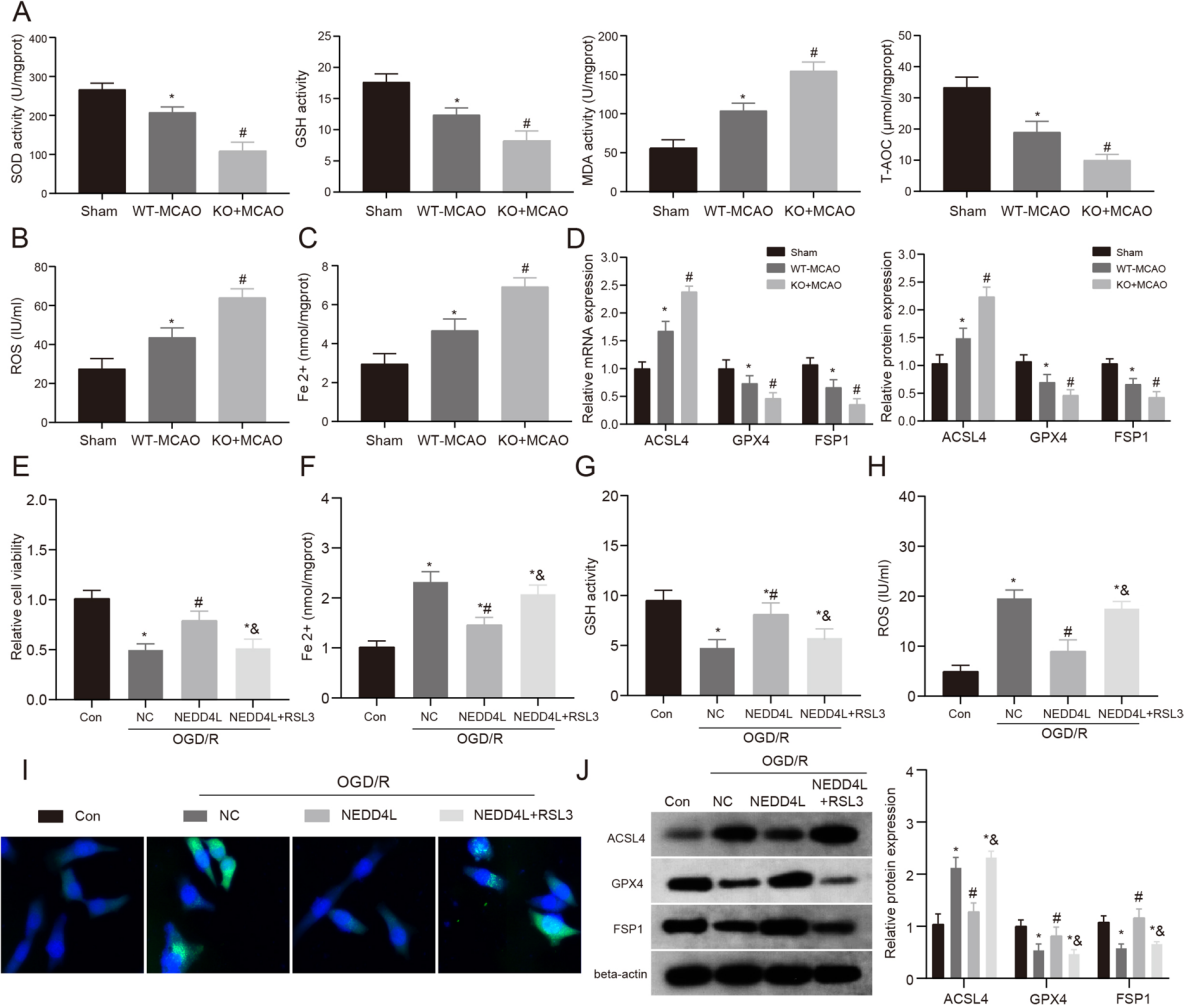
The regulatory role of NEDD4L in ferroptosis during MCAO and OGD/R models. **A**. In this study, the contents of SOD, GSH, MDA, and T-AOC in each treatment group were detected. **B**. The contents of ROS in each treatment group were detected. **C**. The contents of Fe 2 + in each treatment group were detected. **D**. QRT-PCR and WB were used to detect the expression levels of ferroptosis markers in each treatment group. *n* = 6, * vs Sham group *P* < 0.05, # VS WT-MCAO group *P* < 0.05. **E**. Cell viability was assayed using a CCK-8 kit. **F**. The contents of Fe 2 + in HT-22 of each treatment group were detected. **G**. The contents of GSH in HT-22 of each treatment group were detected. **H**. The contents of ROS in HT-22 of each treatment group were detected. **I**. Confocal imaging of BODIPYC11-ROS was performed in HT-22 cells treated with 1 μM RSL3. **J**. WB was used to detect the expression levels of ferroptosis markers in HT-22 of each treatment group. *n* = 3, * vs Con group *P* < 0.05, # vs NC + OGD/R group *P* < 0.05, & vs NEDD4L + OGD/R group *P* < 0.05

Ferroptosis is a form of regulated cellular demise characterized by the accumulation of iron and lipid oxidation. Emerging evidence suggests ferroptosis contributes to neuronal death following ischemic stroke. In this investigation, we investigated the regulatory role of NEDD4L in ferroptosis using both in vivo MCAO and in vitro OGD/R models. We first examined the levels of SOD, GSH, MDA, and T-AOC in brain tissues of three groups. We found that MCAO treatment caused oxidative damage in brain tissues, and NEDD4L-KO mice showed more severe injury (Fig. 3A). This trend was similarly verified by ROS expression levels (Fig. 3B). Accumulation of free Fe 2 + is one of the triggers of ferroptosis. Therefore, we measured Fe 2 + levels and found both MCAO and NEDD4L-KO groups had significantly increased Fe 2 + content, with the NEDD4L-KO group being markedly higher than the MCAO group (Fig. 3C). Finally, we examined the ferroptosis marker to verify our hypothesis that NEDD4L deficiency may exacerbate MCAO-induced brain injury by enhancing ferroptosis (Fig. 3D). In summary, our results indicate MCAO induced oxidative stress and iron accumulation in the brain, while NEDD4L knockout further augmented these effects and exacerbated ischemia-induced brain injury. The heightened ferroptosis signaling observed with NEDD4L deletion suggests NEDD4L may play an inhibitory role in regulating ferroptosis after ischemic stroke.

GPX4 is a glutathione-dependent selenoperoxidase. Its inhibition can promote lipid peroxide accumulation and induce ferroptosis. Therefore, we used the GPX4 inhibitor RSL3 to induce ferroptosis in HT-22 cells and examined the role of ferroptosis in NEDD4L overexpression-improved OGD/R injury. To confirm the role of NEDD4L in regulating ferroptosis, we treated cells with RSL3 alone or in combination with RSL3 and NEDD4L. The levels of Fe 2 + , GSH, and ROS were measured, and it was found that RSL3 treatment could promote the increase of Fe 2 + , while NEDD4L treatment could inhibit this phenomenon. The inhibitory effect of RSL3 on glutathione (GSH) production, however, was found to be counteracted by NEDD4L overexpression. We will further verify its biological function (Supplementary Fig. 2A-C). First, our CCK-8 results showed that NEDD4L overexpression improved OGD/R-induced cytotoxicity in HT-22 cells, but this effect was reversed by the addition of RSL3 (Fig. 3E). Finally, we examined oxidative damage and ferroptosis levels and similarly found that RSL3 could reverse the improvement effects of NEDD4L overexpression on OGD/R-induced oxidative damage and ferroptosis (Fig. 3F–H). In addition, we found the same trend by fluorescence measurement of cellular ROS levels by BODIPYC11-ROS (Fig. 3I). Finally, we examined the protein expression of ACSL4, GPX4, and FSP1 and found that NEDD4L reversed OGD/R-induced ferroptosis, which was reversed by RSL3 (Fig. 3J). Our findings suggest that the inhibitory effects of NEDD4L on ischemic injury are mediated, at least partly, through suppressing ferroptosis.

### NEDD4L regulates ferroptosis in ischemic stroke by controlling TFRC ubiquitination

**Fig. 4:**
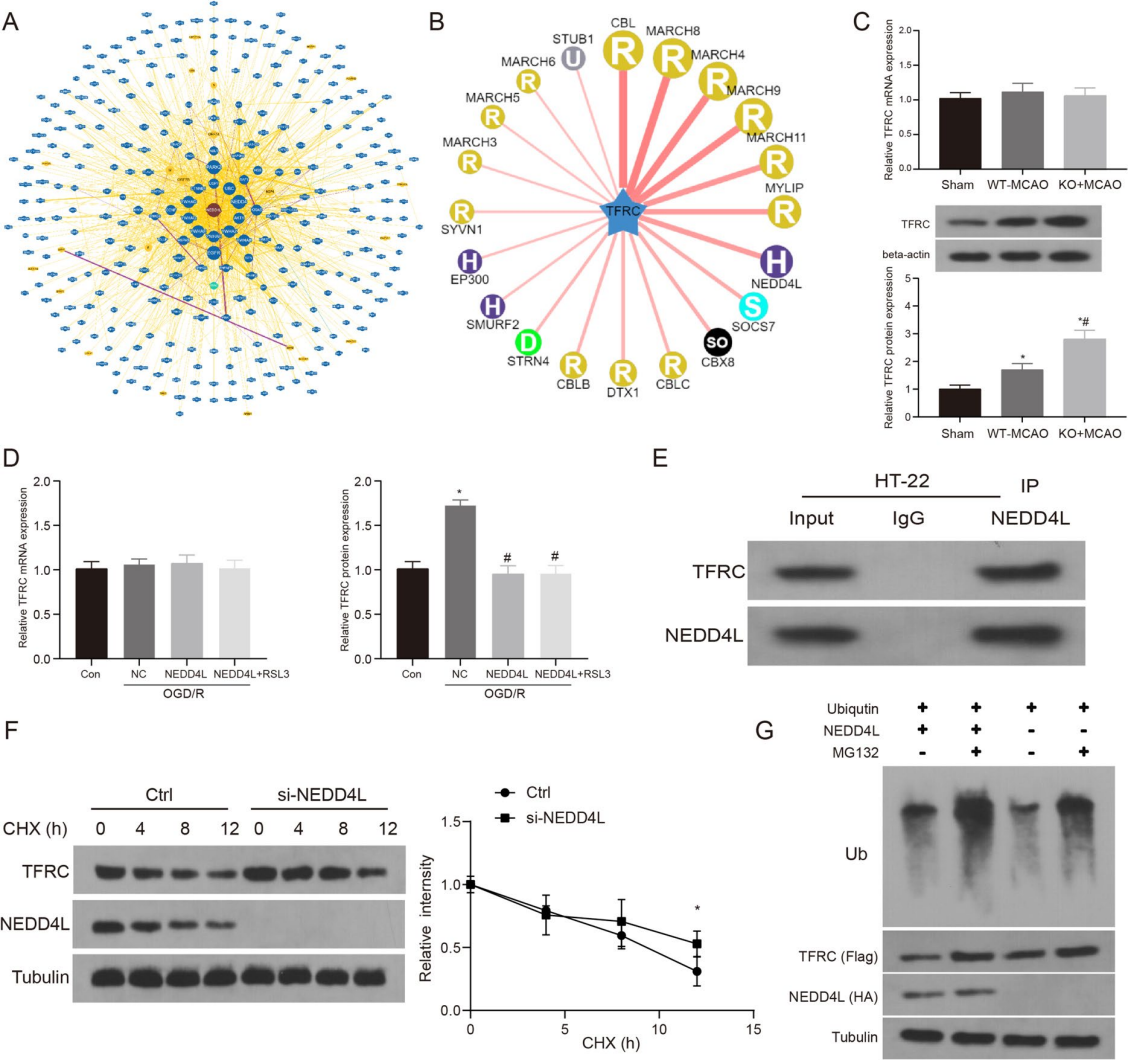
NEDD4L regulates ferroptosis in ischemic stroke by controlling TFRC ubiquitination. **A**. The BioGRID database was used to predict the protein interaction network of NEDD4L. **B**. Ubibroswer database was used to predict TRFC-associated ubiquitinated E3 ligases. **C**. QRT-PCR and WB were used to detect the mRNA and protein expression levels of NEDD4L after MCAO treatment, *n* = 6. **D**. QRT-PCR or WB was used to detect the mRNA expression levels of NEDD4L after OGD/R or NEDD4L treatment, *n* = 3. **E**. CO-IP was used to verify the binding of NEDD4L and TFRC in HT-22 cells. **F**. In this study, a low expression model of NEDD4L was constructed by siRNA technology and then treated with CHX for 0, 4, 8, and 12 h, respectively, to detect the expression of TFRC and NEDD4L proteins to evaluate the protein half-life. **G**. TFRC protein ubiquitination was detected after MG132 (10 μM) treatment

Ferroptosis is a form of regulated cell death modulated by iron levels in ischemic stroke. Our previous work revealed the E3 ubiquitin ligase NEDD4L as a novel regulator of ferroptosis in stroke models. However, the downstream mechanisms by which NEDD4L controls ferroptosis remain unclear. To explore the role of NEDD4L, we first analyzed the BIOGRID database and found that TFRC can interact and bind with NEDD4L (Fig. 4A). Moreover, studies have reported TFRC is an iron carrier protein responsible for internalizing iron bound to transferrin, playing an important role in maintaining brain iron homeostasis and regulating ferroptosis. Our UbiBrowser database showed NEDD4L may be involved in TFRC ubiquitination (Fig. 4B). Therefore, to test our hypothesis, we first examined TFRC expression in MCAO and OGD/R models and found significant changes in TFRC protein levels corresponding to changes in NEDD4L levels in NEDD4L-KO and NEDD4L overexpression groups, while mRNA levels did not significantly differ (Supplementary Fig. 1B) (Fig. 4C–D). Furthermore, co-immunoprecipitation showed NEDD4L and TFRC interact in HT-22 cells (Fig. 4E). Additionally, CHX half-life detection revealed that TFRC half-life was significantly prolonged when NEDD4L was inhibited (Fig. 4F). Finally, we verified NEDD4L regulates TFRC ubiquitination and expression through experiments showing NEDD4L controls TFRC ubiquitination (Fig. 4G).

**Fig. 5:**
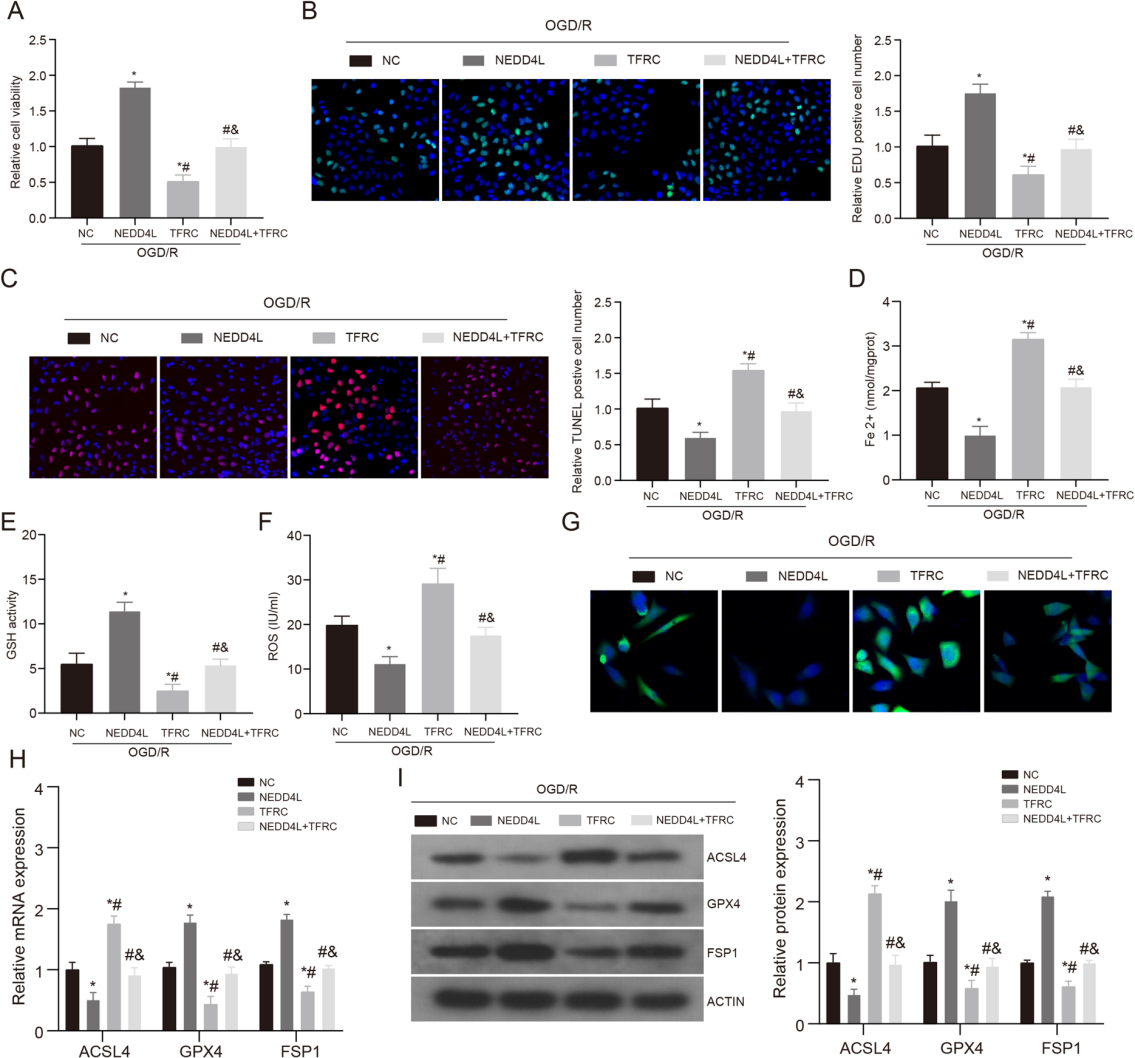
The NEDD4L/TFRC pathway affects cell damage and ferroptosis. **A**. CCK-8 was used to detect the cell viability after NEDD4L or TFRC treatment, *n* = 3. **B**. The Edu staining was used to detect the cell viability after NEDD4L or TFRC treatment, *n* = 3. **C**. The TUNEL staining was used to detect the cell apoptosis after NEDD4L or TFRC treatment, *n* = 3. **D**. The contents of Fe 2 + in HT-22 of each treatment group were detected. **E**. The contents of GSH in HT-22 of each treatment group were detected. **F**. The contents of ROS in HT-22 of each treatment group were detected. **G**. Confocal imaging of BODIPYC11-ROS was performed in HT-22 cells treated with NEDD4L or TFRC. **H**. QRT-PCR was used to detect the expression levels of ferroptosis markers in HT-22 of each treatment group. *n* = 3. **I**. WB was used to detect the expression levels of ferroptosis markers in HT-22 of each treatment group. *n* = 3. * vs NC + OGD/R group *P* < 0.05, # vs NEDD4L + OGD/R group *P* < 0.05, & vs TFRC + OGD/R group *P* < 0.05

To elucidate the regulatory role of TFRC in NEDD4L, we generated NEDD4L and TFRC overexpression models through cell transfection to validate their respective biological (Supplementary Fig. 1C-D). CCK-8 and EdU staining results showed that TFRC overexpression exacerbated cell injury and apoptosis (Fig. 5A–C). However, when NEDD4L and TFRC were treated together, it reversed the improvement effects of NEDD4L on OGD/R. To verify the role of ferroptosis in this process, we detected oxidative damage and ferroptosis markers in these groups. Consistent with the above results, TFRC aggravated OGD/R-induced ferroptosis and NEDD4L’s improvement effects on ferroptosis (Fig. 5D–I). In summary, our findings indicate TFRC overexpression worsens OGD/R-induced neuronal injury and ferroptotic cell death. The protective effects of NEDD4L against ischemic damage appear to be dependent on its ability to degrade TFRC and suppress ferroptosis. These results reveal TFRC as a key effector through which NEDD4L regulates ferroptosis and suggest targeting the NEDD4L-TFRC interaction may have therapeutic potential for mitigating iron-mediated brain injury after stroke.

### METTL3 promotes the mRNA stability of NEDD4L

**Fig. 6:**
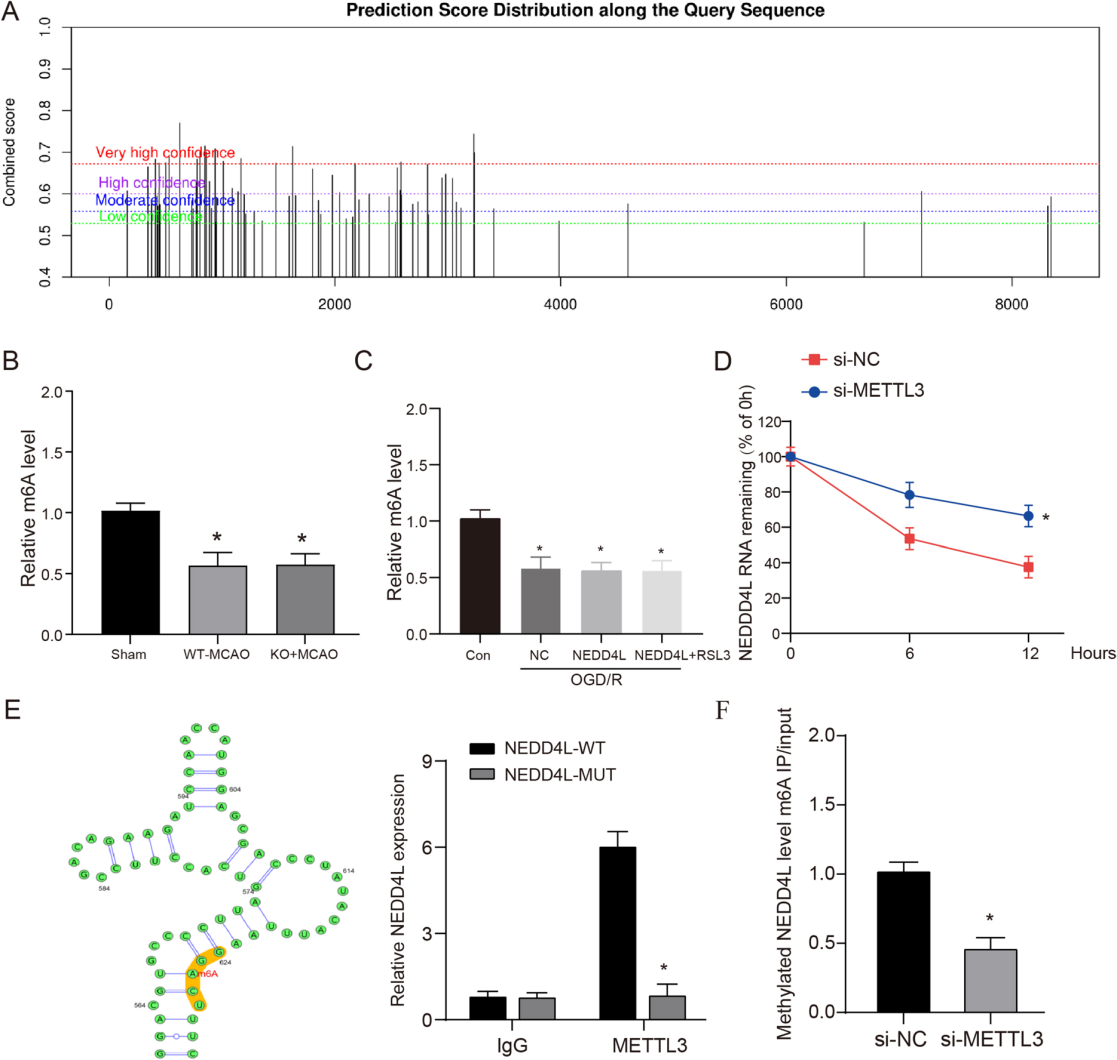
METTL3 promotes the mRNA stability of NEDD4L. **A**. RM2Target and SRAMP data were used to predict the methylation sites of NEDD4L. **B–C**. ELISA was used to detect the expression level of m6A in MCAO model and HT-22 cells. **D**. NEDD4L degradation in si-NC (siRNA-NC was transfected) group and si-METTL3 (siRNA-METTL3 was transfected) group after cycloheximide treatment. **E**. The RIP analysis showing the enrichment of NEDD4L on IgG and METTL3 in the NEDD4L-WT and NEDD4L-MUT. **F** The methylated RNA immunoprecipitation (meRIP) was used to verify the effect of inhibition of METTL3 on NEDD4L m6A levels. Each assay was repeated three times independently. *n* = 3. * vs si-NC group or Con group *P* < 0.05

METTL3 is a writer protein in the m6A process that can stabilize mRNA expression. Therefore, we predicted the NEDD4L methylation sites using the RM2Target and SRAMP databases. The RM2Target database showed METTL3 could bind and regulate NEDD4L (Supplementary Fig. 2). Further results from the SRAMP database revealed NEDD4L has multiple m6A binding sites (Fig. 6A). This indicates m6A regulation may be a key mechanism for METTL3-mediated control of NEDD4L expression. Thus, we examined m6A levels in MCAO tissues and OGD/R cells and found increased m6A levels (Fig. 6B–C). RNA degradation assays showed that inhibition of METTL3 expression could decrease NEDD4L mRNA degradation (Fig. 6D). Finally, we identified and verified that the 564–624 site on NEDD4L is likely the primary binding site for METTL3 (Fig. 6E–F). These data reveal an m6A-dependent regulatory mechanism whereby METTL3 binds to NEDD4L mRNA, installs m6A modifications, and enhances NEDD4L mRNA stability, adding a new layer of post-transcriptional control over this E3 ligase. Defining this METTL3-NEDD4L axis advances our understanding of how m6A signaling finely tunes NEDD4L expression.

### The protective effects of METTL3 overexpression in the MCAO model

**Fig. 7:**
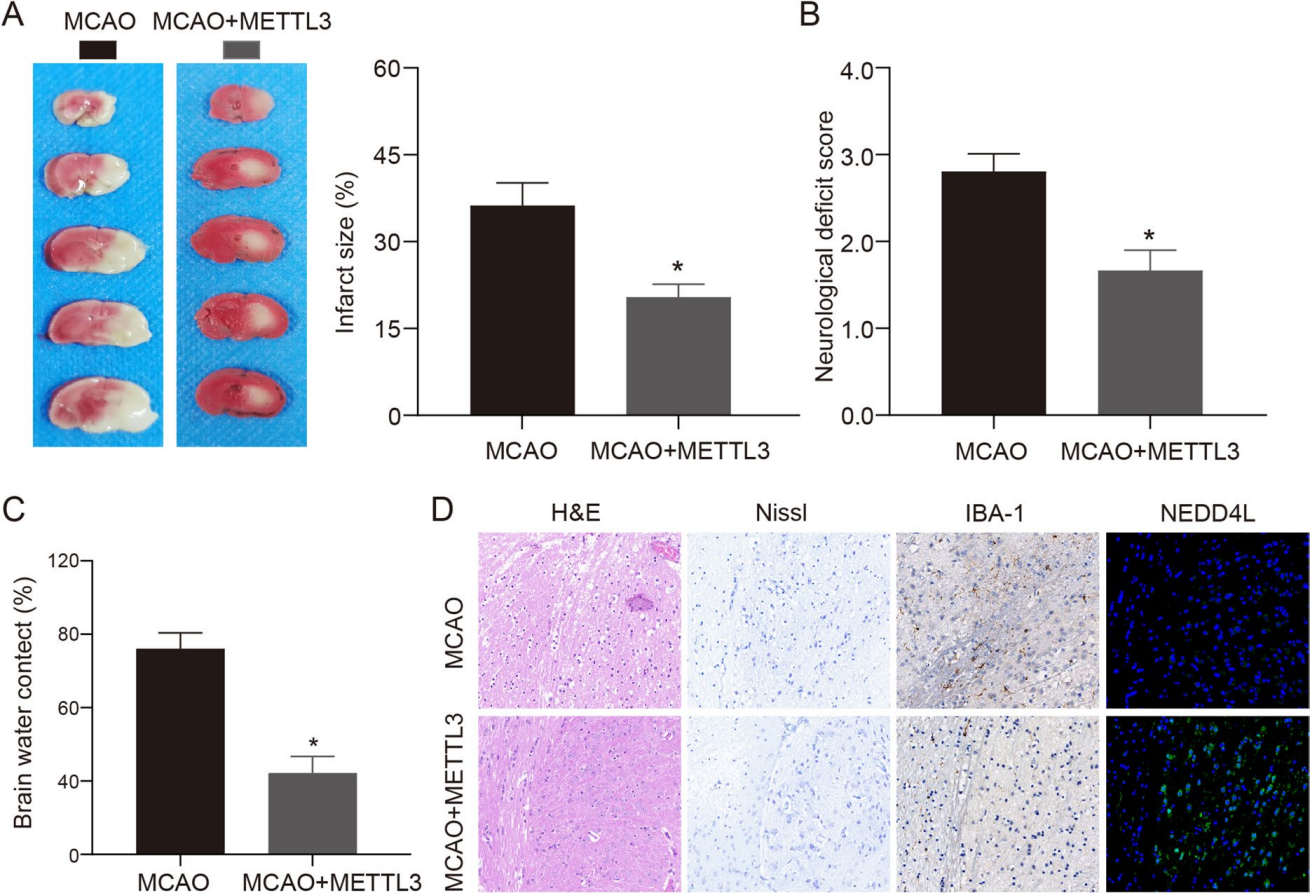
The protective effects of METTL3 overexpression in the MCAO model. **A**. TTC staining was used to detect the ischemic area of mice after MCAO treatment, *n* = 6. **B**. Neurological function scores of mice in each treatment group, *n* = 6. **C**. Brain water content of mice in each treatment group, *n* = 6. **D**. HE, Nissl, immunohistochemistry, and immunofluorescence were used to detect the neuronal damage and inflammatory response in the brain tissue of the two groups. * vs MCAO group *P* < 0.05

**Fig. 8:**
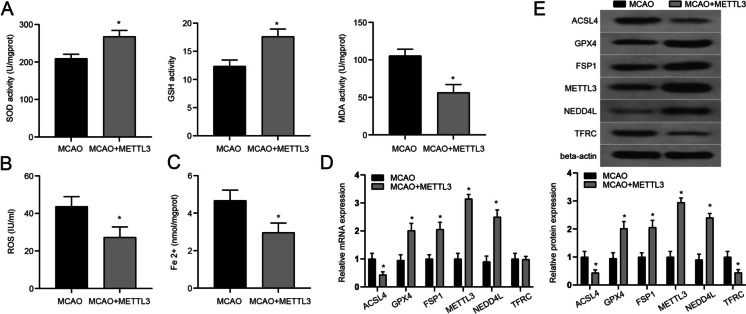
Effect of METTL3 on oxidative damage and ferroptosis. **A**. In this study, the contents of SOD, GSH, and MDA in each treatment group were detected. **B**. The contents of ROS in each treatment group were detected. **C**. The contents of Fe 2 + in each treatment group were detected. **D–E**. QRT-PCR and WB were used to detect the expression levels of ferroptosis markers in each treatment group. *n* = 6, * vs MCAO group *P* < 0.05

Given the regulatory role of METTL3 in promoting NEDD4L expression revealed in our earlier experiments, we next explored whether enhancing METTL3 levels could mimic the beneficial effects of NEDD4L against ischemic injury in vivo. We generated a lentiviral construct to overexpress METTL3 and delivered it by intracerebroventricular injection prior to MCAO surgery in mice. Compared to control MCAO mice, METTL3-overexpressing mice exhibited significantly reduced infarct volume, brain edema, neurological deficits, and oxidative stress. These neuroprotective effects were associated with upregulated NEDD4L levels in the METTL3 overexpression group (Fig. 7). Similarly, we examined oxidative damage and ferroptosis-related markers in brain tissues. We found that METTL3 overexpression could improve MCAO-induced ferroptosis levels through the NEDD4L/TFRC axis (Fig. 8). In summary, our data indicate that the protective effects of METTL3 against MCAO-induced brain injury are mediated through upregulation of NEDD4L and subsequent inhibition of TFRC and ferroptosis. Augmenting METTL3 represents a novel approach to suppress iron-mediated oxidative damage and regulated cell death after ischemic stroke.

## Discussion

Ischemic stroke causes devastating brain injury, and new therapeutic targets are urgently needed. Our previous work revealed the m6A methyltransferase METTL3 promotes expression of the E3 ubiquitin ligase NEDD4L. As NEDD4L suppresses ferroptosis and iron-mediated neuronal death after stroke by ubiquitinating the iron transporter TFRC, we explored whether boosting METTL3 could protect the brain against ischemia through modulating the NEDD4L-TFRC pathway. In mouse stroke models, we found METTL3 overexpression increased NEDD4L levels, enhanced TFRC ubiquitination and degradation, limited iron accumulation, and reduced oxidative damage and ferroptotic cell death, resulting in smaller infarct sizes and improved neurological function. These neuroprotective effects of METTL3 were dependent on NEDD4L, as they were abolished by NEDD4L knockdown. In summary, our study reveals a novel METTL3-NEDD4L-TFRC regulatory axis that suppresses iron-induced neuronal death after ischemic stroke. Augmenting this pathway via METTL3 represents a promising therapeutic strategy to attenuate ferroptosis and preserve the brain after stroke. Our findings shed new light on m6A-mediated gene regulation in cerebral ischemia.

TFRC is an iron carrier protein responsible for internalizing iron bound to transferrin, playing an important role in maintaining brain iron homeostasis and regulating ferroptosis (Yi et al. 2022). When the brain experiences ischemia and hypoxia, expression of HIF-1α increases. HIF-1α can promote transcription of Hmox1 as well as expression of TFR1 and DMT-1. The Hmox1 gene encodes the stress-induced enzyme HO-1, which can degrade hemoglobin to produce Fe 2 + and increase LIP Fe 2 + content, making cells more sensitive to ferroptosis (Lu et al. 2021). HIF-1α mediated upregulation of TFR1 and DMT-1 may be another important factor contributing to iron overload in cerebral IRI. During cerebral ischemia, excitotoxicity increases TF-TFR1 mediated endocytosis, so more transferrin brings iron into endosomes, releasing more iron into the acidic endosomal lumen (Pei et al. 2022). Under oxygen and glucose deprivation, neuronal DMT-1 levels double, and the DMT-1 increase will promote more iron release from endosomes into the neuronal cytoplasmic matrix. These research findings indicate that regulation of TFR1, DMT-1, and HO-1 expression and related TF-TFR1 endocytic pathways are important events affecting intracellular iron overload. This suggests that the iron transport proteins TFRC is aberrantly induced after ischemic brain injury and contributes to toxic iron accumulation through multiple mechanisms, representing potential therapeutic targets (Wang et al. 2022). Further research into the complex molecular regulation of iron homeostasis in cerebral ischemia is still needed.

We had identified TFRC as a new target for the E3 ubiquitin ligase NEDD4L. NEDD4L binds to TFRC and mediates its ubiquitination, leading to quicker degradation. By controlling TFRC levels, NEDD4L can reduce iron uptake and suppress iron-dependent oxidative cell death pathways like ferroptosis after stroke. These findings advance our understanding of how NEDD4L controls iron metabolism through ubiquitination and proteasomal regulation of critical iron-handling proteins. Targeting the NEDD4L-TFRC interaction may represent a promising approach to guard against iron-induced neuronal damage following ischemic events.

The regulation of gene expression is achieved through a significant post-transcriptional modification known as RNA methylation (Chelmicki et al. 2021). Recent findings suggest that RNA methylation plays a significant role in the pathological mechanisms underlying stroke (Chen et al. 2021b). One of the major mechanisms by which RNA methylation regulates stroke is by modulating messenger RNA (mRNA) stability. Methylation of mRNA can increase or decrease its stability, thereby increasing or decreasing protein production from that mRNA (Wang et al. 2020). Therefore, RNA methylation levels are dynamically regulated after stroke, impacting mRNA stability and protein expression, which can either exacerbate brain injury or promote recovery. Modulating RNA methylation may be a promising therapeutic approach, and increased understanding of its regulatory mechanisms in stroke will facilitate the development of RNA methylation-based biomarkers and treatments. At present, several studies have shown that METTL3 has the potential to regulate ANXA-mediated immune microenvironment after stroke (Si et al. 2020; Liu et al. 2023). Our data reveals an m6A-dependent regulatory mechanism whereby METTL3 binds to NEDD4L mRNA, installs m6A modifications, and enhances NEDD4L mRNA stability, adding a new layer of post-transcriptional control over this E3 ligase. Defining this METTL3-NEDD4L axis advances our understanding of how m6A signaling finely tunes NEDD4L expression. Specifically, we demonstrated that lentiviral delivery of METTL3 in mouse brains provided protection against MCAO-induced ischemic injury, reducing infarct volume, brain edema, oxidative damage, and neurological deficits. The neuroprotective effects of METTL3 overexpression were associated with increased NEDD4L expression and decreased TFRC levels in brain tissues, consistent with METTL3 upregulation of NEDD4L and subsequent TFRC downregulation. While previous studies have partially confirmed the involvement of METTL3 in stroke regulation, our study is the first to elucidate the regulatory mechanism of METTL ferroptosis pathway and its association with ubiquitination. Importantly, this discovery also enhances the potential for future targeted therapies and holds significant clinical implications. Taken together, these in vivo data definitively confirm that METTL3 mediates neuroprotection in cerebral ischemia through modulating the NEDD4L-TFRC axis to suppress iron-induced ferroptosis. While our experimental results clearly demonstrate a protective role for the METTL3-NEDD4L-TFRC regulatory axis against ischemic brain injury in mouse models, additional clinical research is needed to confirm the translational relevance of these findings in human stroke patients. The lack of supporting clinical data represents a major limitation of the current study that will need to be addressed going forward. Future priorities will include conducting clinical investigations to verify whether modulating the identified pathways can improve outcomes in human ischemic stroke. We will focus our efforts on gathering patient data to determine if targeting METTL3 and downstream ubiquitination mechanisms confers similar neuroprotection in the clinic as observed in preclinical studies. Addressing these knowledge gaps through human trials will be essential for assessing the therapeutic potential of strategies centered on the METTL3-NEDD4L-TFRC axis against cerebral ischemia–reperfusion injury.

In conclusion, our study provides compelling in vivo evidence that enhancing METTL3 represents a novel therapeutic approach to counteract ischemic brain damage by inhibiting ferroptotic cell death via regulation of iron metabolism. Targeting this newly defined METTL3-NEDD4L-TFRC pathway warrants further investigation as a promising strategy against iron-mediated injury in stroke.

## Supplementary Information

Below is the link to the electronic supplementary material.

Supplementary file1 (JPG 239 KB) QRT-PCR was used to verify the expression levels of NEDD4L and TFRCmRNA in the different models

Supplementary file2 (JPG 133 KB) Regulation of ferroptosis by NEDD4L

Supplementary file3 (JPG 1413 KB) The RM2Target database shows the relationship between METTL3 and NEDD4L and the m6A binding site of NEDD4L

Supplementary file4 (DOCX 16 KB)

## Data Availability

The datasets used and/or analyzed during the current study are available from the corresponding author on reasonable request.
