# Supplementary figures and images for: METTL3 regulates TFRC ubiquitination and ferroptosis through stabilizing NEDD4L mRNA to impact stroke
Source: Cell Biol Toxicol. 2024 Feb 2;40(1):8. doi: 10.1007/s10565-024-09844-x (PMC10834616; doi:10.1007/s10565-024-09844-x)

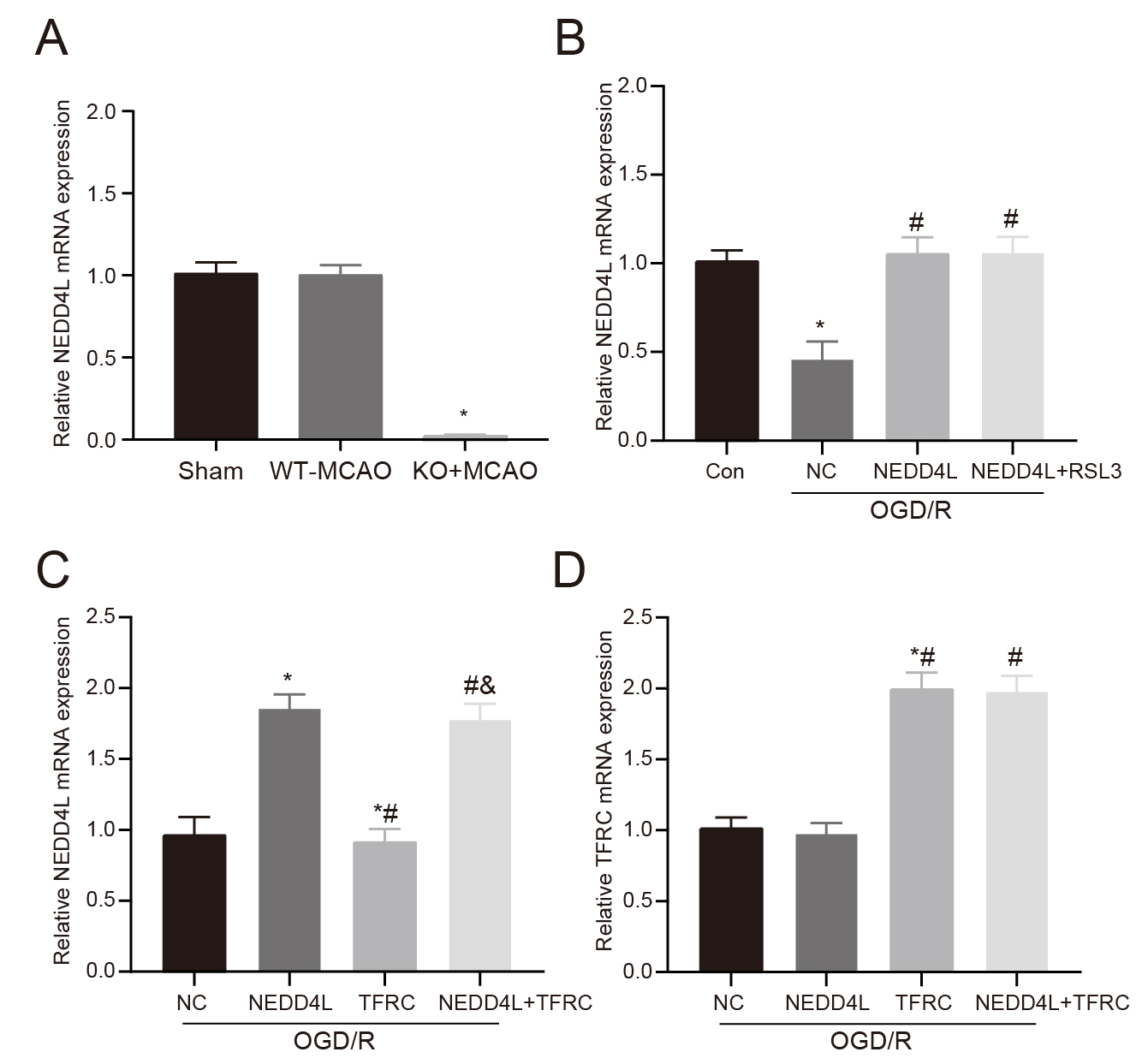

Supplement: Supplementary file 1 — Supplementary file1 (JPG 239 KB) QRT-PCR was used to verify the expression levels of NEDD4L and TFRCmRNA in the different models [file 10565_2024_9844_MOESM1_ESM.jpg]

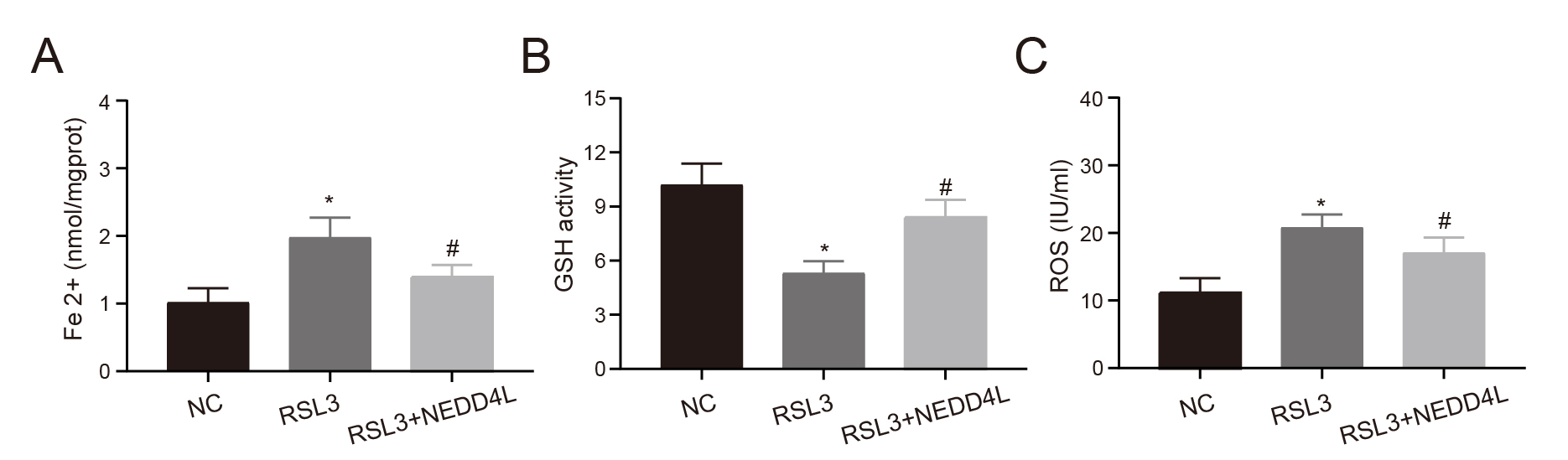

Supplement: Supplementary file 2 — Supplementary file2 (JPG 133 KB) Regulation of ferroptosis by NEDD4L [file 10565_2024_9844_MOESM2_ESM.jpg]

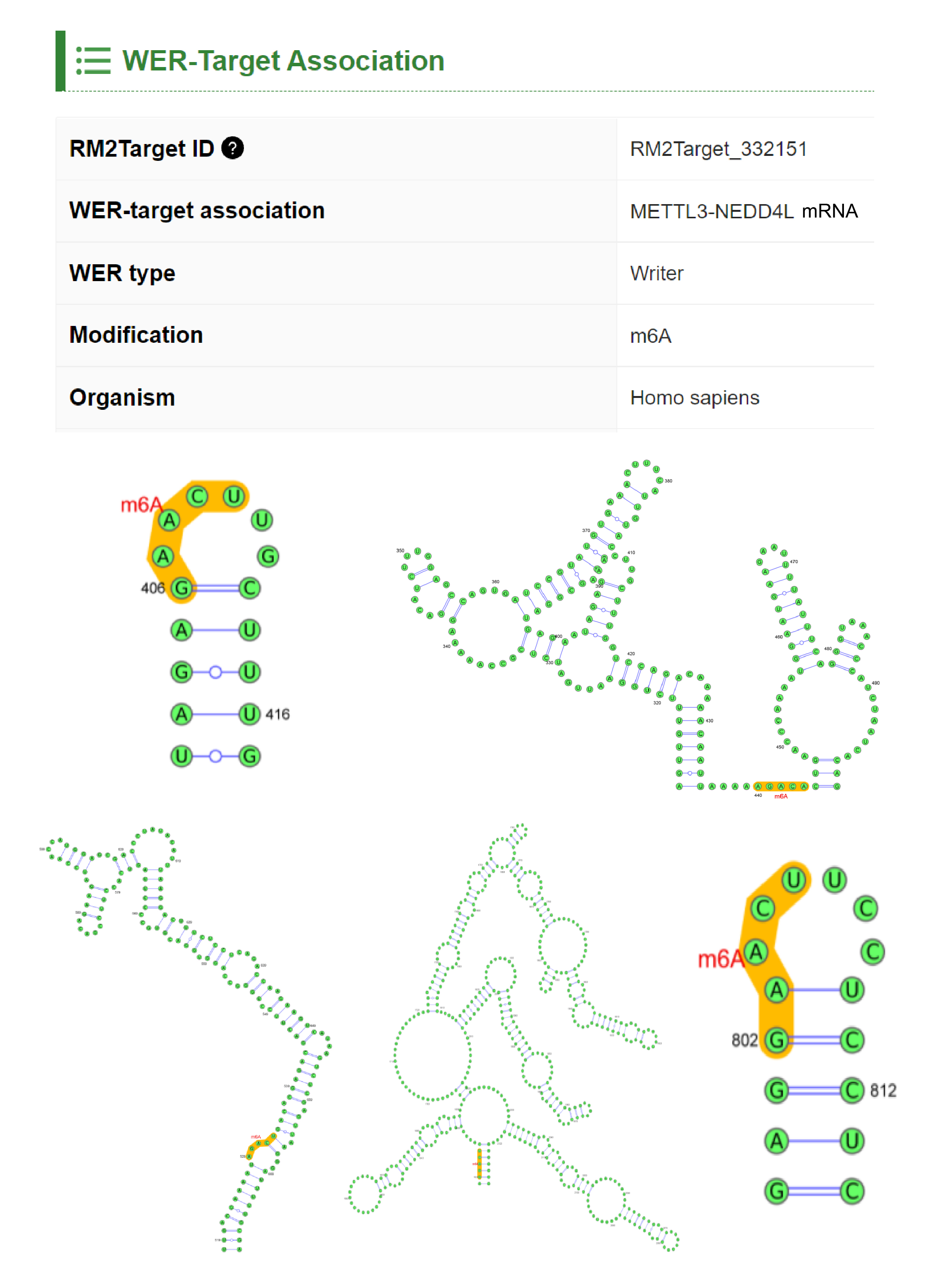

Supplement: Supplementary file 3 — Supplementary file3 (JPG 1413 KB) The RM2Target database shows the relationship between METTL3 and NEDD4L and the m6A binding site of NEDD4L [file 10565_2024_9844_MOESM3_ESM.jpg]
