# Supplementary material for: METTL3 regulates TFRC ubiquitination and ferroptosis through stabilizing NEDD4L mRNA to impact stroke
Source: Cell Biol Toxicol. 2024 Feb 2;40(1):8. doi: 10.1007/s10565-024-09844-x (PMC10834616; doi:10.1007/s10565-024-09844-x)
Supplement: Supplementary file 4 — Supplementary file4 (DOCX 16 KB) [file 10565_2024_9844_MOESM4_ESM.docx]

**Supplementary Table 1: the primer sequences of qRT-PCR**

| Gene symbol | Forward sequence (5’-3’) | Reverse sequence (5’-3’) |
| --- | --- | --- |
| ACSL4 | ACTGGCGATATTGGAGAAT | CACATAGGACTGGTCACTT |
| GPX4 | CAGGAGCCAGGAAGTAAT | CAGCCGTTCTTATCAATGAG |
| FSP1 | CAAGTGGCCCTGGCTGACAA | TGGCCACCTCTGTGCCTTTG |
| TFRC | ACCATTGTCATATACCCGGTTCA | CAATAGCCCAAGTAGCCAATCAT |
| NEDD4L | CAACTTGGACTCGGCCAATC | GTTACTGTTGGCGAGCTGAG |
| β-actin | AGATCAAGATCATTGCTCCTCCT | ACGCAGCTCAGTAACAGTCC |
